# Validation of the Central Auditory Processing Self-Perception Questionnaire (QAPAC) applied to students, parents, and teachers

**DOI:** 10.1590/2317-1782/e20250121en

**Published:** 2026-05-18

**Authors:** Beatriz Lopes Tambascia, Nádia Giulian de Carvalho, Maria Francisca Colella-Santos, Maria Isabel Ramos do Amaral

**Affiliations:** 1 Departamento de Desenvolvimento Humano e Reabilitação, Universidade Estadual de Campinas – UNICAMP - Campinas (SP), Brasil.

**Keywords:** Hearing, Auditory Perception, Child, Screening, Questionnaire

## Abstract

**Purpose:**

To analyze the performance of children with and without academic difficulties, as well as that of their parents and teachers, on the Central Auditory Processing Self-Perception Questionnaire (QAPAC), and to assess the sensitivity and specificity of the questionnaire for the diagnosis of Central Auditory Processing Disorder (CAPD).

**Methods:**

A total of 257 children from a public school, aged between six and ten years, without diagnoses of cognitive impairments or neurodevelopmental disorders, participated in the study. Stage 1, conducted at the school, included peripheral hearing screening and the administration of the QAPAC. Stage 2, conducted at the Institution’s laboratory, involved basic audiological evaluation and behavioral assessment of central auditory processing (CAP), as well as administration of the QAPAC to parents and distribution of the teacher version to educators. At the end of data collection, the sample was divided into group 1 (G1) – 171 children without complaints and/or academic difficulties, and group 2 (G2) – 86 children with academic difficulties. The mean scores were compared within and between groups. Based on G1 performance, receiver operating characteristic (ROC) curve analysis was performed to determine cutoff points for normal performance, risk, and high risk of CAPD.

**Results:**

G1 showed significantly better performance than G2 on all three versions of the QAPAC (p<0.005). In G2, teachers reported worse scores compared to the children (p<0.001). The cutoff points established for CAPD risk were 44.5 for children's responses and 42.5 for teachers, considering the best balance between sensitivity and specificity.

**Conclusion:**

Children with academic difficulties showed poorer performance on the QAPAC, regardless of the respondent. The sensitivity and specificity values support the use of QAPAC as a valid screening tool for central auditory processing.

## INTRODUCTION

Language- and learning-related processes are complex and depend, in part, on the integrity of the auditory system, both peripheral and central^(1)^. Auditory disorders, including Central Auditory Processing Disorder (CAPD), are common in school age^(2)^. CAPD is defined as a deficit in the neural processing of auditory stimuli not attributable to peripheral hearing loss, reflecting dysfunction within the Central Auditory Nervous System (CANS) that can lead to difficulties in the auditory mechanisms of sound localization, auditory discrimination, and auditory pattern recognition, in addition to the perception of pauses or intervals between stimuli, dichotic listening, and sound recognition under adverse conditions, such as background noise or degraded sounds^(3,4)^.

School settings are particularly challenging for listening, and children with CAPD often have difficulty understanding spoken language in case of noisy conditions or rapid speech. These problems can affect the ability to follow complex auditory commands and distinguish words with similar sounds, with significant impact on learning^(5)^.

Traditionally, School Hearing Screening (SHS) is geared toward early detection of peripheral hearing losses, especially due to the high incidence of otitis media in this age group. More recent studies note the importance of including validated procedures that also assess central auditory skills, facilitating early detection and intervention in children at risk of CAPD, minimizing impacts on academic and social development^(6-8)^. Efficient referral for diagnostic assessment enables early identification and timely intervention, thereby minimizing the adverse academic and social consequences associated with CAPD^(9-11)^.

The inclusion of questionnaires as complementary tools in Central Auditory Processing (CAP) screening protocols has been recommended by both national and international guidelines^(3,4,12,13)^. The questionnaires are low-cost and easy to administer, consisting of questions related to everyday situations that require significant performance in auditory skills. Therefore, they provide a qualitative assessment of auditory perception and behavior^(14,15)^. These tools can be answered by individuals being assessed and by their parents and teachers, in addition to other people who are part of the child's daily life, which enables building a broader view on the scope and severity of hearing problems in different scenarios^(14)^.

In the context of CAP screening, the adoption of questionnaires can assist in obtaining information that supports the need for referral for diagnostic assessments, based on a risk score. Moreover, they can function as methods capable of showing the functional impact of CAPD on the child's life. Thus, the application and guidelines involving the use of questionnaires have the potential to constitute preventive work, in addition to assisting in the development of strategies that promote improved auditory skills and trace functional impacts before and after auditory training^(15)^.

In the international literature, the development of computerized programs aimed at screening auditory skills is observed^(16).^ In the Brazilian context, the online program AudBility was designed for screening auditory skills^(17)^. The program is divided into different modules adapted for each age group and consists of auditory tasks, in addition to three versions of the Central Auditory Processing Self-Perception Questionnaire (QAPAC), to be applied with children, parents, or teachers. The QAPAC was designed through adaptations based on the validated questionnaire “Scale of Auditory Behavior- SAB”^(15)^. The initial studies for validation of AudBility analyzed the performance of students aged six to eight years on a battery of six auditory tasks from the program and correlated them with diagnostic behavioral tests. The results showed adequate task efficacy values, but without a focus on the QAPAC questionnaire and its different application methods proposed by the program^(9,18-20)^. A study was conducted and applied the QAPAC to students aged 8 to 12 years with and without learning difficulties, demonstrating a positive and moderate correlation with the sample's performance in the Simplified Auditory Processing Assessment (ASPA)^(21)^. Despite the results demonstrating the instrument's good performance in differentiating the groups, it is important to conduct correlation analyses with the CAPD diagnostic test set, providing the questionnaire with validation data.

Given the above, this study aimed to analyze the results of applying QAPAC in a sample of children with and without learning difficulties and compare both groups as to responses of children and responses of parents and teachers in the respective versions of the instrument. Furthermore, based on the data obtained, considering the group of participants without academic difficulties and the sensitivity and specificity study, we established application criteria based on the cutoff points to be considered in relation to the risk and/or occurrence of CAPD.

## METHOD

### Study type, location, and ethical aspects

This descriptive, prospective, cross-sectional study was conducted in collaboration with a public school in the state of São Paulo and the institution’s Audiology Laboratory. The project was approved by the Institution's Research Ethics Committee, under opinion No. 2,294,609. Participation in the study was restricted to individuals who voluntarily agreed to participate and who signed the informed consent form. In addition, an assent form was obtained for the children involved.

### Participant selection and inclusion and exclusion criteria

Initially, an invitation letter was sent to the parents of all children enrolled from the 1st to the 5th grade. The study included boys and girls, aged six to ten years, and native speakers of Brazilian Portuguese. Children with prior diagnoses of cognitive disorders/syndromes or other conditions affecting neuropsychomotor and/or linguistic development, as per school records, were excluded from the study.

This study was divided in two stages, which will be described below.

### Stage 1 – Auditory skills screening

The procedures were conducted by a speech-language pathologist experienced in the field, in a quiet room provided by the school. The following procedures were performed:

- External auditory canal visual inspection: to check the presence or absence of cerumen. Children with total obstruction were referred for removal and subsequently re-assessed.
- Immittance audiometry (Interacoustics MT10 model equipment): to check middle ear integrity and function. Tympanometric curve and ipsilateral acoustic reflex were performed. Only children with tympanometric curves of type A, Ar, or Ad and the presence of reflexes were included. Children with alterations (curves B or C) were referred to a physician and, subsequently, re-assessed^(22)^.

After ensuring ideal assessment conditions, the online auditory skills screening program -AudBility- was applied. The program features auditory tasks related to skills of sound localization, temporal resolution, temporal ordering, figure-ground for verbal sounds, auditory closure, and dichotic listening with digits. In addition, this instrument includes three versions of the QAPAC geared toward students, parents, and teachers. During the school screening, the children answered the instrument for students, with the researcher's assistance in marking the answers on the computer screen.

This study conducted no analysis of the students' performance on the auditory screening tasks included in the program; we considered only the QAPAC score, which will be described below:

- Central Auditory Processing Self-Perception Questionnaire - QAPAC: it was designed based on the instrument “*Scale of Auditory Behaviors*” (SAB), validated in the European Portuguese version^(15)^. In the program's version, the SAB's twelve original sentences were transformed into direct questions, aiming for better participant comprehension, and a situation was added before each question, with the objective of contextualizing the question within the child's experience/life and facilitating understanding. SAB statement six (6), which refers to difficulty in reading and writing, was modified to a question related to difficulty with sound localization, considering that item nine (09) already addresses the occurrence of school and learning difficulties. The questionnaire follows a scale-based response format, referring to the frequency with which the event or difficulty occurs, namely: always (one point), frequently (two points), sometimes (three points), rarely (four points), and never (five points). As a modification in relation to the SAB, the AudBility program was added with pictographic resources related to each response possibility on the computer screen, in an attempt to aid and motivate the child. The score for each item was tabulated, as well as the total score, which can range from 12 to 60 points. Chart 1 presents the description of the questions in each of the studied versions.

**Chart 1:** Questionnaires based on the Scale of Auditory Behaviors – child, parent, and teacher version

**Child Version**	**Parent Version**	**Teacher Version**
**Response options** □	**Always (1 point) / Frequently (2 points) / Sometimes (3 points) / Rarely (4 points) / Never (5 points)**	
1. You are in a classroom or setting where people are talking.	1. When your child is in a setting where people are talking.	1. When your student is in a setting where people are talking.
**Do you have difficulty hearing or understanding what the teacher is saying?**	**Do they have difficulty hearing or understanding what people are saying?**	**Do they have difficulty hearing or understanding what people are saying?**
		
2. The teacher or a person is speaking very fast to you.	2. If you speak very fast to your child.	2. If you speak very fast to your student.
**Do you have difficulty understanding what the teacher said?**	**Do they have difficulty understanding what was said?**	**Do they have difficulty understanding what was said?**
		
3. The teacher or a person is giving you spoken instructions (explanations).	3. When you give your child spoken instructions (explanations).	3. When you give your student spoken instructions (explanations).
**Do you have difficulty following the spoken instructions?**	**Do they have difficulty following the spoken instructions?**	**Do they have difficulty following the spoken instructions?**
		
		
4. The teacher or a person is speaking to you in a quiet setting.	4. If you are speaking to your child in a quiet setting.	4. If you are speaking to your student in a quiet setting.
**Do you have difficulty hearing and understanding the words clearly without swapping any letters?**	**Do they have difficulty hearing and understanding the words clearly without swapping any letters?**	**Do they have difficulty hearing and understanding the words clearly without swapping any letters?**
		
5. When the teacher or a friend is speaking to you.	5. When you are speaking to your child	5. When you are speaking to your student.
**Do you feel like sometimes you hear well and sometimes you don't?**	**Do you feel like sometimes they hear well and sometimes they don't?**	**Do you feel like sometimes they hear well and sometimes they don't?**
6. You are in the classroom or in the schoolyard and someone calls your name.	6. When your child is called by name in a large setting.	6. When your student is in the classroom or in the schoolyard and someone calls them by name.
**Do you have difficulty perceiving where the sound is coming from?**	**Do they have difficulty perceiving where the sound is coming from?**	**Do they have difficulty perceiving where the sound is coming from?**
7. The teacher or a person is speaking to you.	7. When you are speaking to your child.	7. When you are speaking to your student.
**Do you ask them to repeat what was said?**	**Do they ask you to repeat what was said?**	**Do they ask you to repeat what was said?**
		
8. You are in the classroom.	8. When your child is at home or in other settings.	8. When your student is in the classroom.
**Do you get distracted easily?**	**Do they get distracted easily?**	**Do they get distracted easily?**
		
9. Last year at school.	9. Last year at school.	9. Last year at school.
**Did you have difficulty learning?**	**Did your child have difficulty learning?**	**Did your student have difficulty learning?**
		
10. You are doing an activity.	10. When your child is doing a school activity.	10. When your student is doing an activity in the classroom.
**Do you have difficulty paying attention?**	**Do they have difficulty paying attention?**	**Do they have difficulty paying attention?**
		
11. When you are in the classroom or at home.	11. When your child is at home.	11. When your student is in the classroom.
**Do people say you are a daydreamer or inattentive?**	**Do you think they are a daydreamer or inattentive?**	**Do you think they are a daydreamer or inattentive?**
12. When you are at school or at home.	12. When your child is at home.	12. When your student is in the classroom.
**Are you disorganized?**	**Are they disorganized?**	**Are they disorganized?**

### Stage 2 – Diagnostic stage

All children who passed stage 1 were invited to participate in the second stage, and those whose parents agreed to attend the Audiology Laboratory underwent the Complete Basic Audiological Assessment (Threshold Tonal Audiometry, Speech Audiometry, and Immittance Audiometry) and the Behavioral Central Auditory Processing Assessment. The exams were performed in an acoustic booth with an AC40 – Interacoustics audiometer, TDH 49 headphones, and an Interacoustics AT226 immittance meter, all duly calibrated.

Basic audiological assessment: composed of otoscopy, threshold tonal audiometry, speech audiometry, and acoustic immittance measures (tympanometry and acoustic reflex testing). The adopted normality criterion considered a tonal average of 500Hz, 1kHz, 2kHz, and 4KHz less than 20dB, compatibility as to speech tests and presence of acoustic reflex from 70 to 100dB above the pure tone audibility threshold, at frequencies from 500 to 4000Hz^(22)^.
Behavioral assessment of Central Auditory Processing (CAP): throughout the assessment, the researchers were attentive to factors related to fatigue, inattention, sleepiness, or other aspects that could interfere with the child's performance. When necessary, two assessment sessions were conducted, and given the alteration of a single test, this procedure was reapplied to confirm the findings, especially considering the six-year-old children. The behavioral tests applied were analyzed according to the normality criteria established in the adopted literature, described below:
  1. Masking Level Difference (MLD) – sound localization and lateralization skill/ binaural interaction mechanism. Normal: >9dB^(23)^.
  2. Dichotic Digit Test (DDT) – figure-ground skill for verbal sounds assessed by the binaural integration mechanism. Normal: 6 years – RE 81% and LE 74%; 7 to 8 years – RE 85% and LE 82%; and from 9 years: 95% in both ears^(24)^.
  3. Pediatric Sentence Identification Test with Ipsilateral Competing Message (PSI) – figure-ground skill for verbal sounds assessed under monaural listening in children aged 6 to 8 years. For children aged >9 years, with adequate reading comprehension, the Synthetic Sentence Identification Test (SSI) was administered. Normal: S/N Ratio: -15dB SL = 60%^(24)^;
  4. Random Gap Detection Test (RGDT) - temporal resolution skill and inter-stimulus gap discrimination mechanism. Normal: 6 years <15ms; 7 to 8 years <10ms^(25)^.
  5. Speech test with noise – auditory closure skill and physical sound distortion mechanism in monaural listening. The performance, under good listening conditions, is expected to be greater than 70% of correct identifications and, under regular listening conditions, to be greater than 50%^(24)^.
  6. Frequency Pattern Test – temporal ordering skill/sound pattern discrimination mechanism. For children aged 6 to 8 years, the test developed by Auditec^(26)^ was applied. In children aged 9 and 10 years, the test developed by Musiek was applied. Normal: 9 years: 65%; 10 years: 72%^(27)^.

CAPD diagnosis was established when at least two diagnostic tests yielded abnormal results, in accordance with current clinical guidelines^(3,4)^. For six-year-old children, there are suggested normality criteria for the selected tests, which were followed. Clinical guidelines suggest that, at this age, the assessment should be interpreted as a result of "risk for CAPD"^(28)^ or "delay in the development of central auditory processing skills"^(29), r^einforcing the need for follow-up for these children^(28)^. That is, regardless of the term to be used, the alterations suggest the need for intervention. Considering that and the objective of the present study of the QAPAC's sensitivity in identifying the alteration, the authors opted for using the terminology "normal" or "CAPD," considering the aforementioned criterion, regardless of participant age.

In stage 2, parents answered a medical history questionnaire to confirm the child's health history and school performance. At this time, the parents also answered the QAPAC – parent version, which was printed and administered with the researcher's assistance, in a room separate from the child.

Finally, the teacher version QAPAC was requested to be answered by the teacher responsible for each of the children, considering only those who completed all stages of the study. This questionnaire version was printed and sent to the school in letter format with a one-week completion deadline.

At the beginning of stage 1, right after the child's participation was confirmed, the teacher responsible for each class answered a questionnaire developed by the researchers that contained questions about each student whose participation in the study had been consented to. The questions addressed the teacher's overall perception of the student's school performance, reading and writing skills, relationships with peers, and possible hearing difficulties or complaints of each child. The collected data were analyzed only at the end of the data collection period, with the objective of dividing the sample participants into two distinct groups for analysis of the results:

Group 1 (G1): Children with no school complaints and school performance considered age-appropriate, as assessed by the responsible teacher.
Group 2 (G2): Children with learning difficulties, also attested by the responsible teacher and confirmed by school records.

The total study sample consisted of 257 children who participated in the first stage, with 171 in G1 and 86 in G2. In G1, 89 children (52.04%) were female and 82 (47.95%) were male. The ages in G1 ranged from 6 years and 0 months to 10 years and 11 months, with a mean age of 8.47+1.45 years. The G2 was composed of 43 children (50%) of each sex, with ages ranging from 6 years and 0 months to 10 years and 11 months, and a mean age of 8.24 +1.26 years. Of the 257 students aged six to ten years, 107 completed all versions of the QAPAC and attended stage 2, with 71 from G1 and 36 from G2.

### Analysis of the results

The collected data were processed using SPSS software, with a significance level set at 5%, and parametric tests were selected after confirmation of the normality distribution of the data.

Initially, there was an intergroup comparative analysis as to sex and age homogeneity, using the Two Proportions Equality and Chi-Square tests for the age range of six to ten years. The QAPAC results were explored through descriptive statistics, including mean, median, standard deviation, minimum and maximum values, covering the answers of children, parents, and teachers for each group.

The ANOVA test compared QAPAC performance across the three studied versions, considering an intragroup and intergroup analysis (G1 and G2). In cases of statistical difference, Bonferroni Multiple Comparison was performed as a post hoc analysis.

Subsequently, the QAPAC sensitivity and specificity were evaluated by calculating the Area Under the ROC Curve (AUC), considering that values above 0.5 indicate significance and the higher the value, the better the diagnostic capacity of the test.

Finally, the cutoff point with the best balance between sensitivity and specificity was considered for a descriptive analysis, comparing the distribution of the groups' performance in the QAPAC (risk *versus* no risk) and the performance in the diagnostic assessment of CAP (normal *versus* abnormal) for children aged six to eight years. Thereafter, the criteria/points for normality, risk, and high risk for CAPD were determined.

## RESULTS

There was no intergroup difference in terms of distribution by sex (p=0.757) and age range (p=0.069). Table 1 presents the comparison of average QAPAC performance between groups G1 and G2, considering the responses of students, parents, and teachers. Group 1 demonstrated significantly better performance than Group 2 in all three versions of the questionnaire (p<0.001).

**Table 1:** Comparison of the average performance obtained in the QAPAC application between G1 and G2, considering each questionnaire version applied to the age group from 6 to 10 years (students, parents, and teachers) (n=257)

**Questionnaire**		**N**	**Mean**	**Median**	**Standard Deviation**	**Min**	**Max**	**p-value**
Students	G1	171	46.19	47	7.33	26	59	**<0.001** ^*^
	G2	86	39.72	40	8.79	18	60	
Parents	G1	98	47.93	50.5	8.63	27	60	<0.001*
	G2	53	36.19	34	11.19	12	56	
Teachers	G1	78	50.09	52	8.81	26	60	<0.001*
	G2	37	30.32	32	12.36	11	59	

* ANOVA

Caption: Min = minimum; Max = maximum

Table 2 illustrates the comparison of final mean scores among children, parents, and teachers within each group, considering only the sample that completed stage 2 (n=107).

**Table 2:** Results of the final average score obtained in the QAPAC application considering the comparison between students, parents, and teachers who completed the entire stage 2 in each studied group separately (G1 and G2) (n=107)

**Questionnaire**		**N**	**Mean**	**Median**	**Standard Deviation**	**p-value**
G1	Students	71	46.83	49	7.45	**0.033** ^*^
	Parents	71	47.58	50	8.63	
	Teachers	71	49.73	52	9.02	
G2	Students	36	40.36	42	8.54	0.002*
	Parents	36	35.67	33.5	11.38	
	Teachers	36	30.08	32	12.45	

* Repeated Measures ANOVA

8 students from the total sample did not obtain response in the parent questionnaire but obtained in the teacher questionnaire

Statistical analysis showed significant differences in the mean scores of the three questionnaires in both groups (G1: p=0.033; G2: p=0.002). Post hoc analysis identified that the differences occurred between responses of students and teachers. In G1, the means were 46.83 for students and 49.73 for teachers (p-value = 0.028). In G2, the means were 40.36 for students and 30.08 for teachers (p-value = 0.001).

Of the 107 children who completed the second stage, 87 were in the six- to eight-year age group, whereas only 20 were aged nine to ten years. Due to this distribution, the sensitivity and specificity analysis included only the 87 children aged six to eight years. Of these, 55 belonged to G1 and 32 to G2. This analysis was performed only with data from student and teacher questionnaires, as there were no significant differences between the responses of children and parents in any of the groups.

Table 3 presents the sensitivity and specificity at each cutoff point for the student and teacher questionnaires, respectively. The most balanced cutoff point for the student questionnaire was 44.5 (sensitivity of 61.8% and specificity of 63.2%); for the teacher questionnaire it was 42.5 (sensitivity of 61.8% and specificity of 78.9%). Children with total scores above these cutoff points were considered to have passed the screening. Children with lower performance presented two classifications, which are: high risk for CAPD referring to scores less than or equal to 33.5 (sensitivity of 77.9% and specificity of 57.9%) in the teacher QAPAC and scores less than or equal to 33.5 (sensitivity of 95.6% and specificity of 36.8%) in the student QAPAC. Finally, risk for CAPD refers to the range from 33.5 to 42.5 in the teacher QAPAC and from 33.5 to 44.5 in the student QAPAC.

**Table 3:** Sensitivity and specificity of the QAPAC questionnaires applied to students and teachers

**Questionnaires**	**Cutoff**	**Sensitivity (%)**	**Specificity (%)**
Student	**33.5** ^*^ 44.5*	95.6 61.8	36.8 63.2
Teacher	33.5* 42.5*	77.9 61.8	57.9 78.9

* Cutoff values ​​determined by the ROC curve.

Focusing on the data from the 55 children aged six to eight years from G1 with good school performance, Table 4 shows the calculation of the area under the ROC curve (AUC) based on the total score of this group, indicating that both curves presented an area above 0.5, with the curve based on the teacher's score being the most accurate.

**Table 4:** Area Under the Curve (AUC) values calculated based on the total QAPAC score obtained from the responses of G1 students and teachers (n=55)

**Questionnaires**	**Area**	**p-value**	**Lower Lim.**	**Upper Lim.**
Student	0.693*	0.011	0.548	0.838
Teacher	**0.762** ^*^	0.001	0.658	0.865

* Roc Curve

Caption: Lim. = Limit

Chart 2 shows the distribution of children aged six to eight years with total scores indicative or not of risk for CAPD, with a higher proportion of G2 students with scores indicating risk and high risk for CAPD, in both questionnaires.

**Chart 2:** Student distribution according to CAPD risk in the QAPAC questionnaires

Group	QAPAC Classification (Students)	QAPAC Classification (Teachers)
G1 (n=55)	Passed: 67.27%	Passed: 74.55%
	Risk: 25.45%	Risk: 7.27%
	High risk: 7.27%	High risk: 18.18%
G2 (n=32)	Passed: 37.5%	Passed: 15.62%
	Risk: 43.74%	Risk: 12.5%
	High risk: 18.75%	High risk: 71.88%

Table 5 compares the distribution of CAPD diagnoses in groups G1 and G2, and the individual classification obtained in the QAPAC as to risk or high risk, with nine (16%) from G1 and 10 (31.25%) from G2. In G1, although the QAPAC was not sensitive to identify the risk in four students with CAPD (7.2%), in G2 all diagnosed students were classified as "at risk" in at least one version of the questionnaires.

**Table 5:** Relation between CAPD diagnosis and below of normal limits questionnaires, considering the studied groups G1 (n=55) and G2 (n=32)

**Groups**	**CAPD**	**QAPAC students**	**QAPAC teachers**
	**N (%)**	**Classification**	**Classification**
**G1 (n=55)**	1 (1.8%)	High risk	High risk
		
	1 (1.8%)	Risk	Risk
		
	1 (1.8%)	Risk	High risk
		
	1 (1.8%)	High risk	Risk
		
	1 (1.8%)	Passed	Risk
		
	4 (7.2%)	Passed	Passed
		
**G2 (n=32)**	4 (12.5%)	High risk	High risk
		
	3 (9.3%)	Risk	High risk
		
	1 (3.1%)	High risk	Risk
		
	2 (6.2%)	Passed	High risk
		

These data show that, even among students with confirmed diagnosis of CAPD, there is variability in the classifications assigned by different respondents. However, it is observed that most participants were identified as "at risk" by at least one version of the QAPAC, reinforcing its applicability as a screening tool, especially when used complementarily.

## DISCUSSION

Considering the homogeneous distribution of sex and age between groups, results showed better performance in the group of children without school complaints compared to the group with school difficulties, in the three studied versions of the QAPAC, with worse teacher perception regarding the responses of children in the group with school difficulties, with no significant differences between the child's and parents' responses. Furthermore, this study also provided instrument validation based on cutoff points to be used clinically and in school screening proposals.

Regarding group differentiation, this finding shows the relation between auditory skills and the learning process and corroborates the findings of another national study, which applied the same self-perception questionnaire to a sample with similar selection criteria, albeit smaller, in order to obtain as a result the better performance of the group without school complaints^(21)^, as well as other studies that relate CAPD and academic difficulties^(30-32)^.

It is known that questionnaires can be applied to obtain data related to listening and learning situations in a child's daily routine, as well as to monitor the results of auditory training programs. However, to support their application in screening or clinical settings, instruments must demonstrate adequate psychometric properties and validated risk classification criteria for CAPD.

Regardless of the type of questionnaire used, the instrument is expected to be sensitive for the screening stage and for differentiating between a risk group or a no-risk group. In the study of Barry et al.^(14)^, the questionnaire “The LIFE: Child Self-Report of Listening Ability” was administered to students, and the questionnaire Teacher’s Evaluation of Auditory Performance (TEAP) was answered by teachers, and both instruments adequately differentiate the group of children with suspected CAPD from the control group. This underscores the expected potential of this measurement method when applied to central auditory processing (CAP) screening, facilitating early identification of children at risk of CAPD and appropriate referral for comprehensive diagnostic evaluation^(33)^.

Thus, the application and guidelines resulting from the use of questionnaires can constitute important preventive work aimed at developing strategies for stimulating auditory skills and providing guidance on compensatory strategies to be used in listening situations in children's daily routine in different contexts^(15)^. These collected data can support the search for strategies that contribute toward the improvement of children's listening performance, not only at school but also at home and in other social contexts.

As for intragroup analysis, we observed that the application with teachers resulted in worse scores compared with the application directly with children and parents (Table 2). This discrepancy can be attributed to the association between CAPD and learning difficulties in the school setting, which leads professionals involved in the process to more frequently observe manifestations that may indicate possible alterations during this period^(34,35)^. Classes taught in this setting require proper functioning of the different auditory mechanisms related to auditory skills for the message to be truly understood, thus having direct influence on the learning process. Thus, the fact that teachers are in contact with numerous children in school settings justifies their different views on the children's auditory behavior, since educators are also in contact with children who present typical development, which makes manifestations related to altered CAP more evident.

The statistical difference found in the comparison between children and teacher questionnaires, in both groups, motivated the sensitivity and specificity analysis, considering only children and teacher responses (Table 3). Furthermore, due to this research focusing on discussing CAP screening, it is understood that parents will not always be present at this stage. Furthermore, since children and parent responses were similar, the comparison between the children's and teachers' perspectives was sufficient for analyzing the application of the studied instrument in the context of school screening.

The literature recommends screening questionnaire sensitivity in correctly identifying individuals with or possibly with CAPD with values above 50%, with the ideal being above 70%, as demonstrated in some instruments^(10,36)^. When analyzing the data referring to the calculation of the area under the ROC curve (AUC), adequate results were observed for both children and teachers, with area values different from 0.5. However, the best curve was based on the teachers' score, which shows a higher specificity value (78.9%). Such data relate to the instrument's ability to correctly identify individuals with typical development of auditory skills. The point of greatest balance between sensitivity and specificity of the QAPAC targeted at teachers presents a sensitivity value greater than 50%, which is considered acceptable, given that the questionnaire is part of a battery, serving as a complementary instrument and not used in isolation.

One approach to validating self-perception questionnaires is to establish correlations with results of behavioral assessment of CAP, instead of being limited to comparing different groups of respondents of this instrument^(14)^. In our findings, it was possible to observe that in G2 all subjects diagnosed with CAPD obtained a performance below the cutoff with the greatest balance between specificity and sensitivity in at least one of the questionnaires studied. In G1, four children were diagnosed with CAPD and did not present a risk classification in any of the applied versions of the QAPAC, with three children being 6 years old and one being 7 years old. This underscores the need for caution when considering the inclusion of questionnaires in a school screening protocol. Studies reinforce the complementary use of these instruments in CAP screening, in association with batteries that directly access the CANS mechanisms and not as an isolated instrument^(3,4,12,13).^ Especially in young children, there is greater variability in the responses obtained by the instrument. This variability may be related to the child's difficulty in actually assessing their own difficulties considering a specific auditory context. As for the teacher, it can be hypothesized that, in these G1 children, since the immature auditory skills identified by the diagnostic assessment are not impacting school performance, the teacher may pay less attention to the auditory behaviors of these children in the classroom.

CAPD can occur in isolation or in comorbidity with different clinical conditions, including learning disorders. However, even in the absence of academic implications, CAPD can significantly affect the individual. As described by Chermak et al.^(5)^, difficulties can involve understanding speech in noisy settings, retaining auditory information, following oral instructions, and sustaining attention to auditory stimuli. These limitations can lead to increased listening effort, fatigue, frustration, and poor performance in everyday communicational situations.

Thus, it was possible to verify the relation between performance on the applied screening instruments and the clinical findings. These results corroborate the study of Nunes et al.^(15)^, which observed a positive correlation between the SAB scores and six of the eight CAP assessment tests applied, affirming the SAB questionnaire's ability to predict individual performance on CAP tests, thus being able to contribute to CAPD screening.

It is important to note that the lack of a formal assessment of the children's school performance for the division of groups may result in a limited selection criterion, favoring more heterogeneous samples as to the children's actual academic performance and the reduction in sensitivity. Despite that, the joint analysis of the teacher questionnaire, school records regarding remedial classes or school knowledge of prior diagnoses, and the anamnesis with parents conducted in stage 2 constituted a triangulation of information.

From screening to diagnostic tests, it is important to have accuracy studies that provide data on the sensitivity and specificity of the instruments applied, as this study proposed. Thus, established standardized criteria provide higher reliability when including the QAPAC in a CAP screening protocol, whether or not in association with other auditory tasks. Although the results showed adequate screening sensitivity in each of the adopted cutoff points and studied QAPAC versions, i.e., above 70%, it is recommended that, whenever possible, the questionnaire be used in conjunction with a broader auditory skill screening protocol. The questionnaire presents the ability to collect information and function as a suitable tool to show the functional impact of CAPD on a child's daily life. Thus, the CAP screening questionnaires are designed to characterize hearing difficulty, but not to diagnose CAPD^(37)^. The findings of this study support broader implementation of the questionnaire within school-based hearing screening programs, contributing to improved identification of children at risk for CAPD and to more informed clinical decision-making and family guidance.

## CONCLUSION

The QAPAC effectively differentiated the groups, demonstrating that children with learning difficulties showed poorer performance, regardless of the respondent (child, parent, or teacher). The distinct pattern observed in teacher responses suggests greater sensitivity to auditory-related difficulties within the classroom context. The establishment of teacher-based QAPAC cutoff points, derived from sensitivity and specificity analyses based on CAPD diagnosis, supports its use as a central auditory processing screening instrument.
