# Supplementary material for: Validation of the Central Auditory Processing Self-Perception Questionnaire (QAPAC) applied to students, parents, and teachers
Source: Codas. 2026 May 18;38(3):e20250121. doi: 10.1590/2317-1782/e20250121en (PMC13193188; doi:10.1590/2317-1782/e20250121en)
Supplement: Translation

# Translation: Validação do Questionário de Autopercepção do Processamento Auditivo Central (QAPAC) aplicado em escolares, pais e professores

**DOI:** 10.1590/2317-1782/e20250121pt

**Authors:** Beatriz Lopes Tambascia, Nádia Giulian de Carvalho, Maria Francisca Colella-Santos, Maria Isabel Ramos do Amaral

**Affiliations:** 1 Departamento de Desenvolvimento Humano e Reabilitação, Universidade Estadual de Campinas – UNICAMP - Campinas (SP), Brasil.

**Keywords:** Audição, Percepção Auditiva, Criança, Triagem, Questionário

## Abstract

**Objetivo:**

Analisar o desempenho de crianças com e sem dificuldades escolares, pais e professores, no Questionário de Autopercepção do Processamento Auditivo Central (QAPAC), e a sensibilidade e especificidade para o diagnóstico do Transtorno do Processamento Auditivo Central (TPAC).

**Método:**

Participaram 257 crianças de uma escola da Rede Pública, idades entre seis e dez anos, sem diagnósticos de alterações cognitivas ou transtornos do neurodesenvolvimento. A Etapa 1, na escola, incluiu triagem auditiva periférica e aplicação do QAPAC. A etapa 2, no laboratório da Instituição, realizou-se avaliação audiológica básica e comportamental do processamento auditivo central (PAC), além da aplicação do QAPAC com os pais e professores. Ao final da coleta de dados, a amostra foi dividida em grupo 1 (G1) - 171 crianças sem queixas e/ou dificuldades escolares e grupo 2 (G2) - 86 crianças com dificuldades escolares e o escore médio foi comparado intra e intergrupos. Com base no desempenho do G1, a curva ROC determinou os pontos de normalidade, risco e risco elevado de TPAC.

**Resultados:**

G1 apresentou desempenho superior ao G2 nas três versões do QAPAC (p<0,005). No G2, os professores obtiveram piores escores em relação às crianças (p<0,001). Os pontos de corte estabelecidos para o risco de TPAC foi de 44,5 para a resposta da criança e 42,5 para professor, considerando o melhor equilíbrio entre sensibilidade e especificidade.

**Conclusão:**

Crianças com dificuldades escolares apresentaram pior desempenho no QAPAC, independente do respondente. A sensibilidade e especificidade do QAPAC validam seu uso como ferramenta de triagem auditiva do PAC.

## INTRODUÇÃO

Os processos envolvendo linguagem e aprendizagem são complexos e dependem, em parte, da integridade do sistema auditivo, tanto periférico quanto central^(1)^. Alterações auditivas, incluindo o Transtorno do Processamento Auditivo Central (TPAC), são comuns em idade escolar^(2)^. O TPAC é definido como uma dificuldade no processamento neural dos estímulos recebidos via audição periférica, sendo compreendido como uma disfunção no Sistema Nervoso Auditivo Central (SNAC) que pode acarretar em dificuldades nos mecanismos auditivos de localização sonora, discriminação e reconhecimento de sons em sequência, bem como na percepção de pausas ou intervalos entre os estímulos, escuta dicótica e reconhecimento de sons sob condições adversas, como a presença de ruído de fundo ou sons degradados^(3,4)^.

Ambientes escolares são particularmente desafiadores para a escuta, e crianças com TPAC frequentemente têm dificuldades em entender a linguagem falada em condições de ruído ou quando a fala é rápida. Estes problemas podem afetar a capacidade de seguir comandos auditivos complexos e distinguir palavras com sons semelhantes, influenciando significativamente o aprendizado^(5)^.

Tradicionalmente, a Triagem Auditiva Escolar (TAE) visa a identificação precoce de perdas auditivas periféricas, especialmente devido à alta incidência de otite média nessa faixa etária. Pesquisas mais recentes ressaltam a importância da inclusão de procedimentos validados que avaliem também as habilidades auditivas centrais, facilitando a identificação e intervenção precoce em crianças com risco de TPAC, minimizando impactos no desenvolvimento acadêmico e social^(6-8)^. Diante do encaminhamento assertivo para a avaliação diagnóstica, é possível identificar e minimizar prejuízos que o TPAC pode acarretar aos processos de desenvolvimento acadêmico e social da criança, a partir de uma intervenção adequada^(9-11)^.

A inclusão de questionários como ferramentas complementares nos procedimentos de triagem do Processamento Auditivo Central (PAC) tem sido recomendada por diferentes diretrizes nacionais^(12)^ e internacionais^(3,4,13)^. Os questionários são de baixo custo e fácil aplicação constituídos por questões relacionadas a situações do cotidiano que exigem um desempenho significativo das habilidades auditivas. Portanto, permitem avaliar a percepção e o comportamento auditivo de forma qualitativa^(14)^. Esses instrumentos podem ser respondidos tanto pelo indivíduo que está sendo avaliado, quanto por seus pais e professores, além de outras pessoas que fazem parte do dia a dia da criança, o que permite a construção de uma visão mais ampla a respeito do alcance e da gravidade dos problemas auditivos em diferentes cenários^(14)^.

No contexto da triagem do PAC, o uso de questionários pode auxiliar na obtenção de informações que subsidiam a necessidade do encaminhamento para avaliações diagnósticas, a partir de uma pontuação de risco. Além disso, podem funcionar como métodos capazes de expor o impacto funcional do TPAC na vida da criança. Dessa forma, a aplicação e as orientações envolvendo o uso de questionários tem potencial para configurar um trabalho preventivo, além de auxiliar na elaboração de estratégias que promovam o aprimoramento das habilidades auditivas e verificar o impacto funcional pré e pós treinamento auditivo^(15)^.

Na literatura internacional, observa-se o desenvolvimento de programas computadorizados voltados à triagem das habilidades auditivas^(16)^. No cenário brasileiro, o programa on-line AudBility foi idealizado para a triagem das habilidades auditivas^(17)^. O programa é dividido em diferentes módulos adaptados para cada faixa etária e é composto por tarefas auditivas, bem como três versões do Questionário de Autopercepção das Habilidades Auditivas (QAPAC), a ser aplicado com a criança, pais ou professor. O QAPAC foi idealizado a partir de adaptações baseadas no questionário já validado “*Scale of Auditory Behavior- SAB*”^(15)^. Os estudos iniciais de validação do AudBility analisaram o desempenho de escolares entre seis e oito anos em uma bateria composta por seis tarefas auditivas do programa e correlacionaram com os testes comportamentais diagnósticos. Os resultados demonstraram valores adequados de eficácia das tarefas, porém sem foco no questionário QAPAC e suas diferentes formas de aplicação propostas pelo programa^(9,18-20)^. Um estudo foi conduzido e aplicou o QAPAC em escolares de 8 a 12 anos com e sem dificuldades escolares, demonstrou correlação positiva e moderada com o desempenho da amostra na Avaliação Simplificada do Processamento Auditivo (ASPA)^(21)^. Apesar dos resultados terem demonstrado bom desempenho do instrumento em diferenciar os grupos, faz-se importante análises de correlação com a bateria de testes diagnósticos do TPAC, possibilitando dados de validação do questionário.

Diante do exposto, este estudo teve como objetivo analisar os resultados da aplicação do QAPAC em uma amostra de crianças com e sem dificuldades escolares, bem como comparar em ambos os grupos as respostas das crianças com as respostas dos pais e professor nas respectivas versões do instrumento. Além disso, a partir dos dados obtidos, considerando o grupo de participantes sem dificuldades escolares e o estudo de sensibilidade e especificidade, foram estabelecidos critérios de aplicação com base nos pontos de corte a serem considerados com relação ao risco e/ou ocorrência do TPAC.

## MÉTODO

### Tipo, local do estudo e aspectos éticos

Estudo descritivo, prospectivo de corte transversal, realizado em colaboração com uma escola da rede pública do Estado de São Paulo e o Laboratório de Audiologia da instituição onde a pesquisa foi conduzida. O projeto foi aprovado pelo Comitê de Ética em Pesquisa da Instituição, sob o parecer Nº 2.294.609. A participação no estudo foi restrita aos indivíduos que concordaram voluntariamente em participar e que assinaram o Termo de Consentimento Livre e Esclarecido (TCLE). Além disso, foi obtido o Termo de Assentimento das crianças envolvidas.

### Seleção dos participantes e critérios de inclusão e exclusão

Inicialmente, uma carta-convite foi enviada aos pais de todas as crianças do 1° ao 5° ano escolar. O estudo incluiu crianças do sexo masculino e feminino, com idades entre seis e dez anos, e falantes nativos do português do Brasil. Foram excluídas do estudo crianças com diagnósticos prévios de alterações cognitivas/síndromes ou outras condições que afetem o desenvolvimento neuropsicomotor e/ou linguístico, conforme registros escolares.

O presente estudo ocorreu em duas etapas, que serão descritas a seguir.

### Etapa 1 – Triagem das habilidades auditivas

Etapa realizada em uma sala silenciosa cedida pela escola, por fonoaudiólogo experiente na área. Os seguintes procedimentos foram realizados:

- Inspeção visual do meato acústico externo: realizada com o intuito de identificar a presença ou não de cerúmen. As crianças que apresentaram obstrução total foram encaminhadas para a remoção e, posteriormente, reavaliadas.
- Imitanciometria (equipamento modelo Interacoustics MT10): realizada com objetivo de verificar a integridade e funcionamento da orelha média. Realizou-se a curva timpanométrica e reflexo acústico ipsilateral. Somente foram incluídas crianças com curva timpanométrica do tipo A, Ar ou Ad e presença de reflexos. Crianças que apresentaram alterações (curvas B ou C) foram encaminhadas para o médico e, posteriormente, reavaliadas^(22)^.

Após a garantia de condições ideais de avaliação, o programa online de triagem das habilidades auditivas -AudBility- foi aplicado. O programa apresenta tarefas auditivas relacionadas às habilidades de localização sonora, resolução temporal, ordenação temporal, figura-fundo para sons verbais, fechamento auditivo, escuta dicótica com dígitos. Além disso, encontra-se, neste instrumento, três versões do QAPAC direcionados aos escolares, pais e professores. No momento da triagem escolar, a criança respondeu ao instrumento direcionado aos escolares, com auxílio do pesquisador para marcação das respostas na tela do computador.

Na presente pesquisa, não foi analisado o desempenho dos escolares nas tarefas auditivas de triagem presentes no programa, apenas foi considerado o escore do QAPAC, que será descrito a seguir:

- Questionário de autopercepção das habilidades auditivas - QAPAC: foi elaborado com base no instrumento “*Scale of Auditory Behaviors*” (SAB), validado na versão para o português europeu^(15)^. Na versão do programa, as doze sentenças originais do SAB foram transformadas em perguntas diretas, visando a melhor compreensão do participante e foi acrescentada uma situação antes de cada pergunta, com o objetivo de contextualizar a pergunta dentro da experiência/vivência da criança e facilitar o entendimento. A afirmativa seis (6) do SAB que se refere a dificuldade na leitura e escrita foi modificada para uma pergunta relacionada a dificuldade de localização sonora, levando em consideração que o item nove (09) já aborda a ocorrência de dificuldades escolares e de aprendizagem. O questionário segue uma forma de resposta baseada na escala, referente a frequência com que o evento ou dificuldade ocorre, a saber: sempre (um ponto), frequente (dois pontos), algumas vezes (três pontos), raramente (quatro pontos) e nunca (cinco pontos). Como uma modificação em relação ao SAB, foram inseridos no programa AudBility recursos pictográficos relacionados com cada possibilidade de resposta na tela do computador, na tentativa de facilitar e motivar a criança. A pontuação em cada item foi tabulada, assim como o escore total que pode variar de 12 a 60 pontos. A descrição das perguntas em cada uma das versões estudadas encontra-se no Quadro 1.

**Quadro 1:** Questionários baseados no *Scale of Auditory Behaviors* – versão criança, pais e professor

**Versão Criança**	**Versão Pais**	**Versão Professor**
**Opções de resposta**	**Sempre (1 ponto) / Frequentemente (2 pontos) / Algumas vezes (3 pontos) / Raramente (4 pontos) / Nunca (5 pontos)**	
1. Você está em uma sala de aula ou em um ambiente em que tem pessoas conversando.	1. Quando seu filho(a) está em um ambiente em que tem pessoas conversando.	1. Quando seu aluno(a) está em um ambiente em que tem pessoas conversando.
**Você tem dificuldade para escutar ou entender o que a professora está falando?**	**Ele(a) tem dificuldade para escutar ou entender o que as pessoas estão falando?**	**Ele(a) tem dificuldade para escutar ou entender o que as pessoas estão falando?**
		
2. A professora ou uma pessoa está falando muito rápido com você.	2. Se você fala muito rápido com seu filho(a).	2. Se você fala muito rápido com seu aluno(a).
**Você tem dificuldade para entender o que a professora falou?**	**Ele(a) tem dificuldade para entender o que foi falado?**	**Ele(a) tem dificuldade para entender o que foi falado?**
		
3. A professora ou uma pessoa está dando instruções (explicações) faladas para você.	3. Quando você dá instruções (explicações) faladas para o seu filho(a).	3. Quando você dá instruções (explicações) orais para seu aluno(a).
**Você tem dificuldade para seguir as instruções faladas?**	**Ele(a) tem dificuldade para seguir as instruções faladas?**	**Ele(a) tem dificuldade em seguir as instruções faladas?**
		
		
4. A professora ou uma pessoa está falando com você em um ambiente silencioso.	4. Se você está falando com seu filho(a) em um ambiente silencioso.	4. Se você está falando com seu aluno(a) em um ambiente silencioso.
**Você tem dificuldade para escutar e entender claramente as palavras sem trocar nenhuma letra?**	**Ele(a) tem dificuldade para escutar e entender claramente as palavras sem trocar nenhuma letra?**	**Ele(a) tem dificuldade para escutar e entender claramente as palavras sem trocar nenhuma letra?**
		
5. Quando a professora ou um amigo está falando com você.	5. Quando você está falando com seu filho (a)	5. Quando você está falando com seu aluno(a).
**Você tem a sensação que às vezes você ouve bem e às vezes não?**	**Você tem a sensação de que ele(a) as vezes ouve bem e as vezes não?**	**Você tem a sensação de que às vezes ele(a) ouve bem e às vezes não?**
6. Você está em sala de aula ou no pátio da escola e alguém chama seu nome.	6. Quando seu filho(a) é chamado pelo nome em algum local amplo.	6. Quando seu aluno(a) está na sala de aula ou no pátio e alguém o chama pelo nome.
**Você tem dificuldade para perceber de onde vem o som?**	**Ele(a) tem dificuldade para perceber de onde vem o som?**	**Ele(a) tem dificuldade para perceber de onde vem o som?**
7. A professora ou uma pessoa está falando com você.	7. Quando você está falando com seu filho (a).	7. Quando você está falando com seu aluno(a).
**Você pede para repetir o que foi falado?**	**Ele(a) pede para repetir o que foi falado?**	**Ele(a) pede para repetir o que foi falado?**
		
8. Você está na sala de aula.	8. Quando seu filho está em casa ou em outros ambientes.	8. Quando seu aluno está na sala de aula.
**Você fica distraído com facilidade?**	**Ele(a) fica distraído com facilidade?**	**Ele(a) fica distraído(a) com facilidade?**
		
9. No ano passado na escola.	9. No ano passado na escola.	9. No ano passado na escola.
**Você teve dificuldades para aprender?**	**Seu filho(a) teve dificuldades para aprender?**	**Seu aluno(a) teve dificuldade para aprender?**
		
10. Você está fazendo uma atividade.	10. Quando seu filho(a) está fazendo uma atividade escolar.	10. Quando seu aluno está fazendo uma atividade na sala de aula.**Ele(a) tem dificuldade para ficar atento?**
**Você tem dificuldade para ficar atento?**	**Ele(a) tem dificuldade para ficar atento?**	
	
11. Quando você está em sala de aula ou em casa.	11. Quando seu filho(a) está em casa.	11. Quando seu aluno(a) está na sala de aula.
**As pessoas falam que você é sonhador ou desatento?**	**Você acha que ele (ela) é sonhador(a) ou desatento(a)?**	**Você acha que ele(a) é sonhador(a) ou desatento(a)?**
12.Quando você está na escola ou em casa.	12. Quando seu filho(a) está casa.	12. Quando seu aluno(a) está na sala de aula.
**Você é desorganizado?**	**Ele(a) é desorganizado?**	**Ele(a) é desorganizado(a)?**

### Etapa 2 – Etapa diagnóstica

Todas as crianças que passaram pela etapa 1 foram convidadas a participar da segunda etapa e aquelas cujos pais concordaram em comparecer ao Laboratório de Audiologia passaram pela Avaliação Audiológica Básica Completa (Audiometria Tonal limiar, Logoaudiometria e Imitanciometria), bem como pela Avaliação Comportamental do Processamento Auditivo Central. Os exames foram realizados em cabina acústica com audiômetro AC40 – Interacoustics, fones TDH 49 e Imitanciômetro Interacoustics AT226, devidamente calibrados.

Avaliação audiológica básica: composta pela Meatoscopia, Audiometria Tonal Liminar, Logoaudiometria e Medidas de Imitância Acústica (Timpanometria e Pesquisa dos Reflexos Acústicos). O critério de normalidade adotado considerou média tonal de 500Hz, 1kHz, 2kHz e 4KHz menor que 20dB, compatibilidade quanto aos testes de fala e presença de reflexo acústico de 70 a 100dB acima do limiar de audibilidade para tom puro, nas frequências de 500 a 4000Hz^(22)^.
Avaliação comportamental do PAC: durante toda a avaliação, os pesquisadores estiveram atentos a fatores relacionados à cansaço, desatenção, sonolência ou outros aspectos que pudessem interferir no desempenho da criança. Quando necessário, foram realizadas duas sessões de avaliação e, diante da alteração de um único teste, este procedimento foi reaplicado para confirmação dos achados, especialmente considerando as crianças de seis anos. Os testes comportamentais aplicados foram analisados segundo os critérios de normalidade estabelecidos na literatura adotada, descritos a seguir:
  1. Teste Limiar Diferencial de Mascaramento (MLD – Masking Level Difference) – habilidade de localização e lateralização sonora/ mecanismo de interação binaural. Normalidade:> 9dB^(23)^.
  2. Teste Dicótico de Dígitos (TDD) – habilidade de figura-fundo para sons verbais avaliada pelo mecanismo de integração binaural. Normalidade: 6 anos – OD 81% e OE 74%; 7 a 8 anos – OD 85% e OE 82% e a partir de 9: 95% em ambas as orelhas^(24)^.
  3. Teste de Identificação de Sentenças Pediátricas com Mensagem Competitiva Ipsilateral (PSI) – habilidade de figura-fundo para sons verbais avaliada sob escuta monótica em crianças de 6 até 8 anos. Para as crianças acima de 9 anos, com domínio de leitura considerado adequado, foi aplicado o Teste de Identificação de Sentenças Sintéticas (SSI). Normalidade: Relação S/R: -15dBNS = 60%^(24)^;
  4. Teste de Detecção de Intervalos Aleatórios (RGDT) - habilidade de resolução temporal e mecanismo de discriminação de pausas inter estímulos. Normalidade: 6 anos <15ms; 7 a 8 anos <10ms^(25)^.
  5. Teste de fala com ruído – habilidade de fechamento auditivo e mecanismo de sons fisicamente distorcidos em escuta monótica. Espera-se que o desempenho, na condição boa de escuta, seja superior a 70% de identificações corretas e, na condição regular de escuta, seja superior a 50%^(24)^.
  6. Teste de Padrão de Frequência– habilidade de ordenação temporal/mecanismo de discriminação de padrões sonoros. Para as crianças de 6 a 8 anos, foi aplicado o teste desenvolvido pela Auditec^(26)^. Já nas crianças de 9 e 10 anos, aplicou-se o teste desenvolvido pelo Musiek. Normalidade: 9 anos: 65%; 10 anos: 72%^(27)^.

O desempenho em cada teste foi tabulado e considerou-se o resultado de TPAC a partir de, no mínimo, dois testes diagnósticos alterados^(3,4)^. Para crianças com seis anos, existem critérios de normalidade sugeridos para os testes selecionados e estes foram seguidos. As diretrizes clínicas sugerem que, nessa idade, a avaliação seja interpretada como um resultado de “risco para o TPAC”^(28)^ ou “atraso do desenvolvimento das habilidades auditivas centrais”^(29), r^eforçando a necessidade de acompanhamento para essas crianças ^28^.Ou seja, independente do termo a ser utilizado, as alterações sugerem a necessidade de intervenção. Considerando isso e o objetivo do presente estudo do estudo da sensibilidade do QAPAC ao identificar a alteração, os autores optaram por utilizar a terminologia de “normal” ou “TPAC”, considerando o critério anteriormente mencionado, independente da idade do participante.

Na etapa 2, os pais responderam a uma anamnese para confirmar o histórico de saúde e desempenho escolar da criança. Neste momento, os pais também responderam ao QAPAC – versão para pais, que foi impresso e a aplicação conduzida com o auxílio do pesquisador, em sala separada da criança.

Finalmente, o QAPAC na versão do professor foi solicitado a ser respondido pelo professor responsável de cada uma das crianças, considerando apenas aquelas que completaram todas as etapas do estudo. Esta versão do questionário foi impressa e enviada para a escola em formato de carta com prazo de preenchimento de uma semana.

No início da etapa 1, logo após a confirmação de participação da criança, o professor responsável de cada turma respondeu a um instrumento elaborado pelos pesquisadores que continha perguntas sobre cada aluno cuja participação havia sido consentida no estudo. As perguntas abordavam a percepção global do professor acerca do desempenho escolar, habilidades de leitura e escrita, o relacionamento com os pares e possíveis dificuldades ou queixas auditivas de cada criança. Os dados coletados foram analisados somente ao final do período de coleta de dados, com o objetivo de dividir participantes amostra em dois grupos distintos para a análise dos resultados:

Grupo 1 (G1): Crianças sem queixas escolares e desempenho escolar considerado adequado para a idade, conforme avaliado pelo professor responsável.
Grupo 2 (G2): Crianças com dificuldades escolares, também atestadas pelo professor responsável e confirmadas por registros escolares.

A amostra total do estudo consistiu em 257 crianças que participaram da primeira etapa, sendo 171 no G1 e 86 no G2. No G1, 89 crianças (52,04%) eram do sexo feminino e 82 (47,95%) do sexo masculino. As idades no G1 variaram de 6 anos e 0 meses a 10 anos e 11 meses, com uma média de idade de 8,47+1,45 anos. O G2 foi composto por 43 crianças (50%) de cada sexo, com idades que variaram de 6 anos e 0 meses a 10 anos e 11 meses e média de 8,24 +1,26 anos. Dos 257 alunos de seis a dez anos, 107 completaram todas as versões do QAPAC e compareceram à etapa 2, sendo 71 do G1 e 36 do G2.

### Análise dos resultados

Os dados coletados foram processados utilizando o software SPSS, com um nível de significância estabelecido em 5% e foram selecionados testes paramétricos, após confirmação da distribuição de normalidade dos dados.

No primeiro momento, realizou-se uma análise comparativa entre os grupos em relação à homogeneidade de sexo e idade, utilizando os testes de Igualdade de Duas Proporções e Qui-Quadrado para a faixa etária de seis a dez anos. Os resultados do QAPAC foram explorados através de estatísticas descritivas, incluindo média, mediana, desvio padrão, valores mínimo e máximo, abrangendo as respostas de crianças, pais e professores para cada grupo.

O teste ANOVA comparou o desempenho do QAPAC nas três versões estudadas considerando uma análise intragrupo e entre os grupos (G1 E G2). Em casos de diferença estatística, procedeu-se com a Comparação Múltipla de Bonferroni como análise post hoc.

Posteriormente, avaliou-se a sensibilidade e especificidade do QAPAC por meio do cálculo referente a Área sob a Curva ROC (AUC), considerando que valores acima de 0,5 indicam significância e quanto maior o valor, melhor a capacidade diagnóstica do teste.

Finalmente, foi considerado o ponto de corte de melhor equilíbrio entre sensibilidade e especificidade para a realização de uma análise descritiva, comparando a distribuição do desempenho dos grupos no QAPAC (risco *versus* não risco) e o desempenho na avaliação diagnóstica do PAC (normal *versus* alterado) para crianças de seis a oito anos. A partir daí, foram determinados os critérios/pontos de normalidade, critério de risco e de risco elevado de TPAC.

## RESULTADOS

Não houve diferença entre os grupos em termos de distribuição por sexo (p=0,757) e faixa etária (p=0,069). Na Tabela 1, apresentamos a comparação do desempenho médio no QAPAC entre os grupos G1 e G2, considerando as respostas dos escolares, pais e professores. Observou-se que o G1 teve desempenho superior ao G2 nas três versões do questionário (p<0,001).

**Tabela 1:** Comparação do desempenho médio obtido na aplicação do QAPAC entre o G1 e G2, considerando cada uma das versões do questionário aplicadas na faixa etária de 6 a 10 anos (escolares, pais e professores) (n=257)

**Questionário**		**N**	**Média**	**Mediana**	**Desvio Padrão**	**Min**	**Max**	**P-valor**
Escolares	G1	171	46,19	47	7,33	26	59	**<0,001** ^*^
	G2	86	39,72	40	8,79	18	60	
Pais	G1	98	47,93	50,5	8,63	27	60	<0,001*
	G2	53	36,19	34	11,19	12	56	
Professores	G1	78	50,09	52	8,81	26	60	<0,001*
	G2	37	30,32	32	12,36	11	59	

* ANOVA

Legenda: Min = mínimo; Max = máximo

A Tabela 2 ilustra a comparação dos escores médios finais entre crianças, pais e professores dentro de cada grupo, considerando apenas a amostra que finalizou a etapa 2 (n=107).

**Tabela 2:** Resultados do Escore médio final obtido na aplicação do QAPAC considerando a comparação entre escolares, pais e professores que completaram toda a etapa 2 em cada um dos grupos estudados separadamente (G1 e G2) (n=107)

**Questionário**		**N**	**Média**	**Mediana**	**Desvio Padrão**	**P-valor**
G1	Escolares	71	46,83	49	7,45	**0,033** ^*^
	Pais	71	47,58	50	8,63	
	Professores	71	49,73	52	9,02	
G2	Escolares	36	40,36	42	8,54	0,002*
	Pais	36	35,67	33,5	11,38	
	Professores	36	30,08	32	12,45	

* ANOVA com Medida Repetida

8 escolares da amostra total não obtiveram resposta no questionário dos pais, mas obtiveram no questionário dos professores

A análise estatística revelou diferenças significativas nos escores médios dos três questionários em ambos os grupos (G1: p=0,033; G2: p=0,002). A análise post hoc identificou que as diferenças ocorreram entre as respostas dos escolares e dos professores. No G1, as médias foram 46,83 para os escolares e 49,73 para os professores (p-valor = 0,028). No G2, as médias foram 40,36 para os escolares e 30,08 para os professores (p-valor = 0,001).

Dentre as 107 crianças que completaram a segunda etapa, 87 estavam na faixa etária de seis a oito anos e apenas 20 na faixa de nove a dez anos. Devido a essa distribuição, a análise de sensibilidade e especificidade foi realizada apenas com as 87 crianças de seis a oito anos. Dessas, 55 pertenciam ao G1 e 32 ao G2. Essa análise foi realizada apenas com os dados dos questionários de escolares e professores, pois não houve diferenças significativas entre as respostas das crianças e dos pais em nenhum dos grupos.

A Tabela 3 apresenta a sensibilidade e especificidade em cada ponto de corte para os questionários de escolares e professores, respectivamente. O ponto de corte mais equilibrado para o questionário dos escolares foi 44,5 (sensibilidade de 61,8% e especificidade de 63,2%); para o dos professores foi 42,5 (sensibilidade de 61,8% e especificidade de 78,9%). Foi considerado que escolares com escore total acima destes pontos de corte passaram na triagem. Já crianças com desempenho inferior apresentaram duas classificações, sendo estas: risco elevado para TPAC referente a escores menores ou iguais 33,5 (sensibilidade de 77,9% e especificidade de 57,9%) no QAPAC professores e escores menores ou iguais a 33,5 (sensibilidade 95,6% e especificidade 36,8%) no QAPAC escolares. Por fim, o risco para TPAC refere-se a faixa de 33,5 até 42,5 no QAPAC professores e 33,5 até 44,5 no QAPAC escolares.

**Tabela 3:** Sensibilidade e especificidade dos questionários do QAPAC aplicados a escolares e professores

**Questionários**	**Corte**	**Sensibilidade (%)**	**Especificidade (%)**
Escolar	**33,5** ^*^	95,6	36,8
	44,5*	61,8	63,2
Professor	33,5*	77,9	57,9
	42,5*	61,8	78,9

* Valores de corte determinados pela Curva Roc

Focando nos dados das 55 crianças de seis a oito anos do G1 com bom desempenho escolar, a Tabela 4 mostra o cálculo da área sob a curva ROC (AUC) com base no escore total desse grupo, indicando que ambas as curvas apresentaram área acima de 0,5, com a curva baseada no escore do professor sendo a mais precisa.

**Tabela 4:** Valores de área sob a curva (AUC), calculados com base no escore total do QAPAC, obtidos pelas respostas dos escolares e professores do G1 (n=55)

**Questionários**	**Área**	**P-valor**	**Lim. Inferior**	**Lim. Superior**
Escolar	**0,693** ^*^	0,011	0,548	0,838
Professor	0,762*	0,001	0,658	0,865

* Curva Roc

Legenda: Lim. = Limite

O Quadro 2 mostra a distribuição de crianças de seis a oito anos com escores totais indicativos ou não de risco para TPAC, revelando uma maior proporção de escolares do G2 com pontuação de risco e risco elevado para TPAC, em ambos os questionários.

**Quadro 2:** Distribuição dos escolares conforme risco de TPAC nos questionários do QAPAC

Grupo	Classificação no QAPAC (Escolares)	Classificação no QAPAC (Professores)
G1 (n=55)	Passou: 67,27%	Passou: 74,55%
	Risco: 25,45%	Risco: 7,27%
	Risco elevado: 7,27%	Risco elevado: 18,18%
G2 (n=32)	Passou: 37,5%	Passou: 15,62%
	Risco: 43,74%	Risco: 12,5%
	Risco elevado: 18,75%	Risco elevado: 71,88%

A Tabela 5 compara a distribuição de diagnósticos de TPAC nos grupos G1 e G2, além da classificação individual obtida no QAPAC quanto ao risco ou risco elevado, sendo nove (16%) do G1 e 10 (31,25%) do G2. No GI, apesar do QAPAC não ter sido sensível para identificar o risco em quatro escolares com TPAC (7,2%), no G2 todos os escolares diagnosticados foram classificados como “risco” em pelo menos uma versão dos questionários.

**Tabela 5:** Relação entre diagnóstico de TPAC e questionários alterados, considerando os grupos estudados G1 (n-55) e G2 (n=32)

**Grupos**	**TPAC**	**QAPAC escolares**	**QAPAC professores**
	**N (%)**	**Classificação**	**Classificação**
**G1 (n=55)**	1 (1,8%)	Risco elevado	Risco elevado
		
	1 (1,8%)	Risco	Risco
		
	1 (1,8%)	Risco	Risco elevado
		
	1 (1,8%)	Risco elevado	Risco
		
	1 (1,8%)	Passou	Risco
		
	4 (7,2%)	Passou	Passou
		
**G2 (n=32)**	4 (12,5%)	Risco elevado	Risco elevado
		
	3 (9,3%)	Risco	Risco elevado
		
	1 (3,1%)	Risco elevado	Risco
		
	2 (6,2%)	Passou	Risco elevado
		

Esses dados evidenciam que, mesmo entre escolares com diagnóstico confirmado de TPAC, há variabilidade nas classificações atribuídas pelos diferentes respondentes. No entanto, observa-se que a maioria dos participantes foi identificada como “risco” por pelo menos uma das versões do QAPAC, reforçando sua aplicabilidade como instrumento de triagem, especialmente quando utilizado de forma complementar.

## DISCUSSÃO

A partir de uma amostra homogênea em termos da distribuição por sexo e faixa etária, os resultados verificaram melhor desempenho no grupo de crianças sem queixas escolares em relação ao grupo com dificuldade escolar, nas três versões estudadas do QAPAC, com pior percepção do professor em relação às respostas das crianças do grupo com dificuldade escolar, sem diferenças significativas entre as respostas da criança e pais. Além disso, esse estudo também possibilitou a validação do instrumento a partir de pontos de cortes a serem utilizados clinicamente e em propostas de triagem escolar.

No que se refere a diferenciação dos grupos, este achado evidencia a relação entre as habilidades auditivas e o processo de aprendizagem e corrobora com os achados de outra pesquisa nacional, que aplicou o mesmo questionário de autopercepção em amostra com semelhantes critérios de seleção, porém de menor número, de modo a obter como resultado o melhor desempenho do grupo sem queixas escolares^(21)^, bem como outros estudos que relacionam TPAC e dificuldades acadêmicas^(30- 32)^.

Sabe-se que os questionários podem ser aplicados para a obtenção de dados relacionados às situações de escuta e aprendizagem no cotidiano da criança, bem como para monitorar os resultados de programas de treinamento auditivo. No entanto, para o uso em contexto de triagem ou clínica, é pressuposto que sejam obtidas medidas psicométricas e dados de validação com relação aos critérios de risco para ocorrência do TPAC.

Independentemente do tipo de questionário utilizado, espera-se que o instrumento seja sensível para a etapa de triagem e para a diferenciação entre um grupo de risco ou não. Na pesquisa de Barry et al.^(14)^ o questionário “*The LIFE: Child Self-Report of Listening Ability*” foi aplicado em escolares e o questionário *Teacher’s Evaluation of Auditory Performance (TEAP)* foi respondido pelos professores e ambos os instrumentos diferenciam adequadamente o grupo de crianças com suspeita de TPAC do grupo controle. Isso ressalta o potencial esperado desse método de mensuração quando aplicado na triagem do processamento auditivo central (PAC), permitindo identificar precocemente crianças em risco de TPAC e o encaminhamento assertivo para avaliação diagnóstica^(33)^.

Sendo assim, a aplicação e as orientações decorrentes do uso de questionários podem compor um trabalho preventivo importante voltado para a elaboração de estratégias de estimulação das habilidades auditivas e orientações quanto a estratégias compensatórias a serem utilizadas nas situações de escuta do cotidiano da criança em diferentes contextos^(15)^. Essas informações coletadas podem favorecer a busca por estratégias que contribuam para a melhora do desempenho de escuta da criança não somente na escola, mas também em casa e em outros contextos sociais.

Com relação a análise intragrupo, observamos que a aplicação feita com o professor resultou em piores escores em comparação com a aplicação realizada diretamente com a criança e seus pais (Tabela 2). Esta discrepância pode ser atribuída à associação entre TPAC e dificuldades de aprendizagem no ambiente escolar, o que leva os profissionais envolvidos no processo a observarem com maior frequência manifestações que podem indicar possíveis alterações durante este período^(34,35)^. As aulas ministradas neste ambiente exigem o bom funcionamento dos diferentes mecanismos auditivos relacionados às habilidades auditivas para que a mensagem seja de fato compreendida, exercendo, portanto, influência direta no processo de aprendizagem. Assim, o fato de o professor estar em contato com grande quantidade de crianças no ambiente escolar, justifica a diferença na sua visão sobre o comportamento auditivo da criança, já que o educador também está em contato com crianças que apresentam desenvolvimento típico, o que torna as manifestações relacionadas às alterações no PAC mais evidentes.

A diferença estatística encontrada na comparação entre o questionário das crianças e dos professores, em ambos os grupos, motivou a análise de sensibilidade e especificidade, considerando apenas a resposta da criança e do professor (Tabela 3). Além disso, devido ao foco de discussão desta pesquisa ser voltado para a triagem do PAC, entende-se que os pais nem sempre estarão presentes nessa etapa. Além disso, visto que as respostas das crianças e dos pais foram semelhantes, a comparação entre a visão da criança e do professor foi suficiente para a análise da aplicação do instrumento estudado no contexto da triagem escolar.

A literatura preconiza sensibilidade no questionário de triagem em identificar corretamente indivíduos com ou possivelmente com TPAC com valores acima de 50%, sendo o ideal acima de 70%, conforme demonstrado em alguns instrumentos^(10,36)^. Ao analisarmos os dados referente ao cálculo da área sob a curva Roc (AUC), verificou-se resultados adequados tanto para crianças quanto professores, com valores de área diferentes de 0,5. No entanto, a melhor curva foi baseada no escore dos professores, que apresenta maior valor de especificidade (78,9%). O dado em questão está relacionado à capacidade do instrumento em identificar corretamente indivíduos com desenvolvimento típico das habilidades auditivas. O ponto de maior equilíbrio entre sensibilidade e especificidade do QAPAC direcionado ao professor apresenta valor de sensibilidade maior que 50%, o que configura um valor aceitável, visto que o questionário faz parte de uma bateria, sendo um instrumento complementar e não utilizado isoladamente.

Uma abordagem para validação de questionários de autopercepção é estabelecer correlações com os resultados da avaliação comportamental do PAC ao invés de se limitar à comparação entre diferentes grupos de respondentes deste instrumento^(14)^. Em nossos achados, foi possível observar que no G2 todos os sujeitos diagnosticados com TPAC obtiveram desempenho inferior ao corte de maior equilíbrio entre especificidade e sensibilidade em pelo menos um dos questionários estudados. No grupo G1, quatro crianças foram diagnosticadas com TPAC e não apresentaram classificação de risco em nenhuma das versões aplicadas do QAPAC, sendo três crianças com 6 anos de idade e uma com 7 anos. Esse dado ressalta a necessidade de um cuidado ao considerarmos a inclusão de questionários em um protocolo de triagem escolar. Estudos reforçam o uso complementar desses instrumentos na triagem do PAC, em associação a baterias que acessem diretamente os mecanismos do SNAC e não como instrumento isolado^(3,4,12,13).^ Especialmente em crianças pequenas, observa-se uma maior variabilidade nas respostas obtidas pelo instrumento. Essa variabilidade pode estar relacionada à dificuldade da criança em, de fato, avaliar suas próprias dificuldades considerando um contexto auditivo específico. Quanto ao professor, pode-se levantar a hipótese de que, nessas crianças do G1, já que as imaturidades em habilidades auditivas identificadas pela avaliação diagnóstica não estão impactando no desempenho escolar, o professor pode se atentar menos aos comportamentos auditivos dessas crianças em sala de aula.

O TPAC pode ocorrer de forma isolada ou em comorbidade com diferentes quadros clínicos, incluindo os distúrbios de aprendizagem. No entanto, mesmo na ausência de implicações acadêmicas, o TPAC pode gerar prejuízos significativos ao indivíduo. Conforme descrito por Chermak et al.^(5)^, as dificuldades podem envolver a compreensão de fala em ambientes ruidosos, retenção de informações auditivas, seguimento de instruções orais e atenção sustentada a estímulos auditivos. Tais limitações podem levar a aumento de esforço para escuta, fadiga, frustração e baixo desempenho em situações comunicativas cotidianas

Com isso, foi possível verificar a relação entre o desempenho nos instrumentos de triagem aplicados e os achados clínicos. Estes resultados corroboram com o estudo de Nunes et al.^(15)^, o qual observou uma correlação positiva entre os escores do SAB e seis dos oito testes da avaliação do PAC aplicados, afirmando a capacidade do questionário SAB de prever o desempenho do indivíduo em testes do PAC, sendo capaz de contribuir para a triagem do TPAC.

É importante destacar que o fato de não ter sido realizada avaliação formal do desempenho escolar das crianças para a divisão dos grupos pode resultar em um critério de seleção limitado, favorecendo amostras mais heterogêneas quanto ao real desempenho acadêmico das crianças e a redução na sensibilidade. Apesar disso, a análise em conjunto do questionário do professor, registros escolares quanto a salas de reforço ou conhecimento escolar de diagnósticos prévios, bem como a anamnese com os pais realizada na etapa 2 constituíram um *crosscheck* de informações.

Desde a triagem até os testes diagnósticos, é importante estudos de acurácia que disponibilizem dados de sensibilidade e especificidade dos instrumentos aplicados, assim como esse estudo se propôs. Dessa forma, a partir de um critério padronizado estabelecido há uma maior confiabilidade ao incluir o QAPAC em um protocolo de triagem auditiva do PAC, em associação ou não com outras tarefas auditivas. Ainda que os resultados tenham mostrado uma sensibilidade para triagem adequada em cada um dos pontos de corte adotados e versões do QAPAC estudadas, ou seja, acima de 70%, recomenda-se que, sempre que possível, o questionário seja utilizado em associação a um protocolo mais amplo de triagem das habilidades auditivas. O questionário apresenta a capacidade de coletar informações e funcionar como ferramenta apta para expor o impacto funcional do TPAC no cotidiano da criança. Assim, os questionários de triagem do PAC são elaborados visando caracterizar a dificuldade auditiva, mas não o diagnóstico do TPAC^(37)^. Levando em consideração os resultados deste estudo, espera-se que o questionário tenha maior inserção no contexto da triagem auditiva escolar, garantindo uma melhor identificação de crianças em risco para o TPAC e facilitando a tomada de decisão clínica e orientação familiar com base em um critério mais objetivo e validado.

## CONCLUSÃO

O QAPAC foi eficaz em diferenciar os grupos e demonstrou que crianças com dificuldades escolares tendem a ter pior desempenho, independente do respondente (criança, pai ou professor). O desempenho diferenciado dos professores indica uma percepção mais atenta das dificuldades. A definição dos pontos de corte do QAPAC dos professores, baseada nos dados de sensibilidade e especificidade para o diagnóstico do TPAC, validam o uso como ferramenta de triagem auditiva do PAC.
